# Antioxidants - a combat against oxidative stress in dementia

**DOI:** 10.1016/j.amsu.2022.104632

**Published:** 2022-09-11

**Authors:** Fakhar Latif, Maria Imran

**Affiliations:** a Department of Internal Medicine, Dow University of Health Sciences, Karachi, Pakistan; b Department of Internal Medicine, Jinnah Sindh Medical University, Karachi, Pakistan

**Keywords:** Dementia, Antioxidants, Alzheimer's disease, Oxidative stress, OS, oxidative stress, ROS, reactive oxygen species, NADPH, reduced nicotinamide adenine dinucleotide phosphate, DNA, deoxyribonucleic acid, HNE, 4-hydroxynonenal

Dementia is a senile neurodegenerative syndrome characterised by a progressive deterioration in cognitive function resulting in memory loss and poor thinking skills. It has become an area of growing concern amongst physicians as the ageing population is estimated to increase two-fold by 2050. Currently, there are 55 million dementia patients around the globe, with an additional 10 million cases being reported annually [1].

Dementia has various subtypes: vascular, Alzheimer's, frontotemporal and Lewy body dementia. Despite differences, these types maintain common pathogenesis through oxidative stress (OS), which occurs due to an imbalance caused by abnormally excessive production of reactive oxygen species or a decrease in antioxidant mechanisms [2]. In this regard, the brain tissue is more prone to damage by oxidative stress because it is composed of easily oxidisable lipids, has a higher consumption rate, and lacks antioxidant defence mechanisms. The brain holds for about 20% of the total body oxygen consumption. As it is rich in oxygen, iron, and polyunsaturated fatty acids, it is a prime substrate for free radical reactions, thus making it vulnerable to injury by free radicals and heightening susceptibility to neurodegenerative disorders owing to oxidative stress. Reactive Oxygen Species (ROS) are a by-product of various sources, including the electron transport chain, NADPH oxidases, and myeloperoxidases. These are eliminated from the body by several antioxidants [3]. Therefore, increased levels of oxidative stress due to ageing in the cranial tissue may be one of the risk factors for dementia.

Several studies have confirmed a causal link between oxidative stress and neurodegenerative disorders. Oxidative stress can result from lipid peroxidation, protein oxidation, and DNA oxidation [2]. Lipid peroxidation in the brain produces cytotoxic substances (e.g. 4-hydroxynonenal, or HNE) that can inhibit glycolysis [4]. It is equally important to note that brains with dementia lack glutathione transferases which can help detoxify these toxicants. This results in the degeneration of neurons in the hippocampus, a region involved mainly with long-term memory [4]. The oxidation of enzymes, such as glutamine synthetase and creatine kinase, can markedly affect the physiology of the brain by causing reduced energy metabolism and enhanced excitotoxicity [2]. It is also known to disrupt the ubiquitin-proteasome pathway, which is involved in misfolded protein degradation [5]. Hence, OS can cause an accumulation of misfolded beta-amyloids, resulting in Alzheimer's disease. DNA damage and improper repair mechanisms contribute to protein misfolding as well as other transcriptional and translational defects, leading to dementia. Some studies indicated the presence of OS-related DNA damage and lipid peroxidation in patients with dementia [6]. A handful of them has also identified mitochondrial dysfunction due to a decrease in cytochrome *c* protein in the mitochondria of patients with dementia [7]. This dysfunction can inflict neuronal damage attributed to the production of reactive oxygen species.

Accordingly, a recent study published in Neurology by Beydoun et al. proposed the use of antioxidants and carotenoids as a possible first-line therapy to curb increased oxidative stress in patients with dementia [8]. It was concluded that vitamins A, C, and E acted cooperatively in their affiliation with Alzheimer's disease and all-cause dementia. With the values of lutein + zeaxanthin and β-cryptoxanthin being statistically significant, there was an inverse relationship between individual and total carotenoid plasma concentration with both neurodegenerative disorders. Furthermore, serum vitamin A and α -carotene opposed all-cause dementia; meanwhile, vitamin A and α-carotene, vitamin E and lycopene, and vitamin A and β-carotene had similar effects on Alzheimer's disease incidence. Even though in a previous study Beydoun et al. hinted at the synergistic action of vitamin E and lycopene, the recent study by Beydoun et al. rejected these by identifying a contradictory interaction between vitamin E and lycopene. Hence, putting forward the idea of an alteration in their collective activity over time.

Antioxidants occur in minute quantities in the cell and help inhibit the oxidation of substances by readily reacting with oxygen, acting as reducing agents and buffering ROS. Similarly, Vitamin E and β-carotene significantly counter the adverse effects of free radicals [9]. α-Tocopherol also subdues carbon-centred lipid radicals that, otherwise, give rise to peroxyl radical post reaction with molecular oxygen and may take part in a radical generation. If failed to get reduced by glutathione reductases to hydroxy fatty acids, lipid hydroperoxides account for a variety of lipid peroxidation products: 2-alkenols, epoxides, and malondialdehyde [10]. The predatory nature of vitamin C for ROS protects against lipid peroxidation products. It also helps with one-electron reduction of lipid hydroperoxyl radical through the vitamin E redox cycle [3]. Furthermore, It was also observed that the decreased functioning of these antioxidants is associated with the elevation of signs of post-ischemic injury [[11], [12], [13]].

The inadequacy of research on the relationship between antioxidants and dementia demands that researchers conduct randomised control trials with larger sample sizes and longer follow-ups. Meta-analysis is also needed to assess the synergistic or antagonistic effect of certain antioxidants on each other. Measurements of the complete or partial impact of antioxidants are also required as time progresses, to ensure their efficacious supplementation. In conclusion, it is of utmost importance that potential therapies for dementia are discovered and tested for potency to avoid the repercussions of an ageing population on society, the economy, and medicine in general. Physicians should prioritise antioxidants to improve the quality of life for those affected by dementia as they are widely available naturally and in supplements. Antioxidants are also affordable, which makes them an appropriate course of action to tackle dementia even in low and middle-income countries.

## Ethics approval

Not Applicable.

## Funding sources

The authors have no sources of funding to declare.

## Author statement

Fakhar Latif – study concept or design, writing, reviewing the paper.

Maria Imran – study concept or design, writing the paper.

## Registration of research studies

Name of the registry:

Unique Identifying number or registration ID:

Hyperlink to your specific registration (must be publicly accessible and will be checked):

## Guarantor

Fakhar Latif.

## Consent

Not Applicable.

## Declaration of competing interest

The authors declare that they have no conflicting interests.
